# Supplementary material for: Fetal temporal sulcus depth asymmetry has prognostic value for language development
Source: Commun Biol. 2023 Jan 27;6:109. doi: 10.1038/s42003-023-04503-z (PMC9883513; doi:10.1038/s42003-023-04503-z)
Supplement: Supplementary file 2 — Supplementary Information [file 42003_2023_4503_MOESM2_ESM.pdf]

Supplementary Information

Additional background information

Supplementary Table 1. Group results and comparison

	Overall study group (n = 38)		Study group with fetal STS quantifications (n = 29)		Comparison between groups
	Mean (SD)	Range	Mean (SD)	Range	T or X^2 (p)
**Background information:**					
Sex (f/m)	14/24		10/19		0.04 (.842)
Gestational age at fetal MRI (weeks)	27.68 (3.57)	20.14 – 35.43	29.06 (2.58)	25.29 – 35.43	1.79 (.079)
Age at test (years)	8.85 (1.98)	6.33 – 13.25	9.10 (2.03)	6.33 – 13.25	-0.61 (.547)
Handedness at test (LI)	-0.72 (0.54)	-1.00 – 0.80	-0.67 (0.59)	1.00 – 0.80	0.38 (.709)
Socioeconomic status at test (7-point-scale)	5.30 (1.54)	1.00 – 7.00	5.18 (1.52)	1.00 – 7.00	0.25 (.804)
Perceptual reasoning at test (z-score)	0.15 (1.14)	-2.46 – 2.88	0.07 (1.14)	-2.46 – 1.80	0.08 (.936)
**Language examinations:**					
Expressive vocabulary (z-score)	0.09 (1.20)	-2.33 – 2.33	-0.15 (1.30)	-2.33 – 2.05	0.53 (.601)
Language comprehension (z-score)	0.45 (1.02)	-1.64 – 2.05	-0.39 (0.97)	-1.64 – 1.88	0.14 (.887)
Verbal fluency (z-score)	-0.43 (0.93)	-2.17 – 1.15	-0.34 (0.83)	-2.05 – 1.15	-0.20 (.839)
Verbal memory (z-score)	-0.07 (0.67)	-1.50 – 1.34	-0.13 (0.67)	-1.50 – 1.04	0.35 (.728)

Note: Student’s t-test was performed to compare groups.

Supplementary Table 2. Association of variables of background information with STS measurements, language abilities, language lateralization, and lateralization of white matter tracts

	GA at fetal MRI	Sex	Age at test	Handedness at test	Socioeconomic background at test	Perceptual reasoning at test
	r (p)	t (p)	r (p)	r (p)	r (p)	r (p)
**STS measurements (n = 29):**						
Left STS depth	**0.68 (.000)***	1.67 (.106)	0.18 (.142)	-0.07 (.722)	-0.03 (.863)	-0.09 (.646)
Right STS depth	**0.65 (.000)***	1.84 (.076)	0.08 (.701)	-0.20 (.316)	-0.09 (.666)	-0.11 (.558)
LI of STS depths	0.12 (.551)	0.38 (.705)	0.15 (.452)	0.07 (.708)	-0.04 (.861)	-0.02 (.937)
**Language abilities (n = 29):**						
Expressive vocabulary	0.13 (.517)	-0.28 (.782)	0.16 (.413)	0.15 (.435)	0.17 (.402)	**0.52 (.004)***
Language comprehension	0.05 (.795)	0.40 (.691)	0.34 (.072)	0.08 (.672)	0.14 (.481)	**0.59 (.001)***
Verbal fluency	0.01 (.945)	-1.52 (.142)	0.29 (.141)	0.04 (.834)	0.21 (.298)	0.38 (.050)
Verbal memory	0.06 (.741)	0.88 (.389)	0.12 (.523)	0.06 (.744)	0.20 (.321)	0.36 (.058)
**Language lateralization (n = 23):**						
LI total	0.12 (.578)	-0.20 (.844)	0.15 (.519)	-0.30 (.163)	-0.10 (.660)	-0.03 (.882)
LI frontal lobes	0.12 (.573)	-0.38 (.712)	0.07 (.739)	-0.24 (.278)	-0.11 (.616)	-0.02 (.929)
LI temporal lobes	0.09 (.671)	-0.09 (.928)	0.19 (.382)	-0.24 (.243)	-0.20 (.380)	0.01 (.961)
LI parietal lobes	0.02 (.935)	-0.24 (.815)	0.28 (.201)	-0.23 (.295)	-0.08 (.715)	-0.24 (.267)
**Lateralization of white matter tracts (n = 25):**						
LI AF (FA)	0.24 (.254)	0.32 (.754)	-0.33 (.108)	-0.15 (.481)	-0.10 (.655)	-0.02 (.935)
LI AF (volume)	0.05 (.800)	0.44 (.661)	-0.20 (.342)	0.39 (.060)	0.27 (.207)	0.07 (.734)
LI AF (number of streams)	0.19 (.377)	0.51 (.618)	-0.21 (.309)	0.26 (.223)	0.13 (.548)	0.03 (.879)
LI SLF I (FA)	0.25 (.231)	-0.17 (.869)	-0.23 (.272)	-0.13 (.538)	-0.23 (.278)	-0.11 (.600)
LI SLF I (volume)	-0.10 (.648)	0.40 (.695)	0.14 (.503)	-0.07 (.751)	**0.44 (.033)**	**0.41 (.042)**
LI SLF I (number of streams)	-0.21 (.317)	0.40 (.694)	0.05 (.823)	-0.11 (.613)	0.34 (.108)	0.34 (.096)
LI SLF II (FA)	0.15 (.472)	**2.74 (.012)**	-0.11 (.594)	0.32 (.133)	**0.47 (.020)**	0.14 (.495)
LI SLF II (volume)	0.02 (.914)	1.60 (.125)	0.05 (.799)	0.11 (.599)	-0.23 (.281)	0.00 (.995)
LI SLF II (number of streams)	0.07 (.752)	1.05 (.304)	-0.03 (.895)	0.15 (.497)	-0.36 (.089)	-0.09 (.655)
LI SLF III (FA)	0.21 (.335)	-0.23 (.823)	-0.39 (.058)	0.19 (.383)	-0.39 (.057)	-0.29 (.159)
LI SLF III (volume)	-0.14 (.510)	0.52 (.606)	0.03 (.878)	0.33 (.120)	0.35 (.091)	**0.41 (.043)**
LI SLF III (number of streams)	-0.12 (.566)	0.18 (.857)	-0.03 (.883)	0.34 (.110)	0.19 (.366)	0.23 (.277)
LI ILF (FA)	0.03 (.902)	0.71 (.488)	0.28 (.179)	0.18 (.400)	0.28 (.190)	0.16 (.460)
LI ILF (volume)	0.16 (.455)	0.31 (.754)	0.08 (.716)	-0.01 (.952)	0.22 (.313)	0.02 (.938)
LI ILF (number of streams)	0.11 (.614)	1.10 (.284)	0.09 (.664)	-0.02 (.911)	0.02 (.324)	-0.04 (.839)
LI UF (FA)	-0.23 (.271)	-1.23 (.233)	-0.08 (.728)	**0.42 (.044)**	-0.14 (.537)	0.20 (.340)
LI UF (volume)	0.04 (.853)	0.22 (.827)	0.13 (.535)	-0.17 (.452)	0.05 (.839)	-0.27 (.206)
LI UF (number of streams)	0.07 (.753)	0.27 (.787)	0.10 (.635)	-0.20 (.349)	0.36 (.087)	-0.17 (.409)

Note: Correlations involving cognitive data, LIs of the STS depths, and DTI measurements were performed with Pearson's correlation. For correlations involving fMRI LIs, Spearman rank correlations were used. STS, superior temporal sulcus; LI, laterality index; AF, arcuate fasciculus; SLF, superior longitudinal fasciculus; ILF, inferior longitudinal fasciculus, UF, uncinate fasciculus; FA, fractional anisotropy. Bold letters indicate $p < .05$, * indicates significance after Bonferroni correction

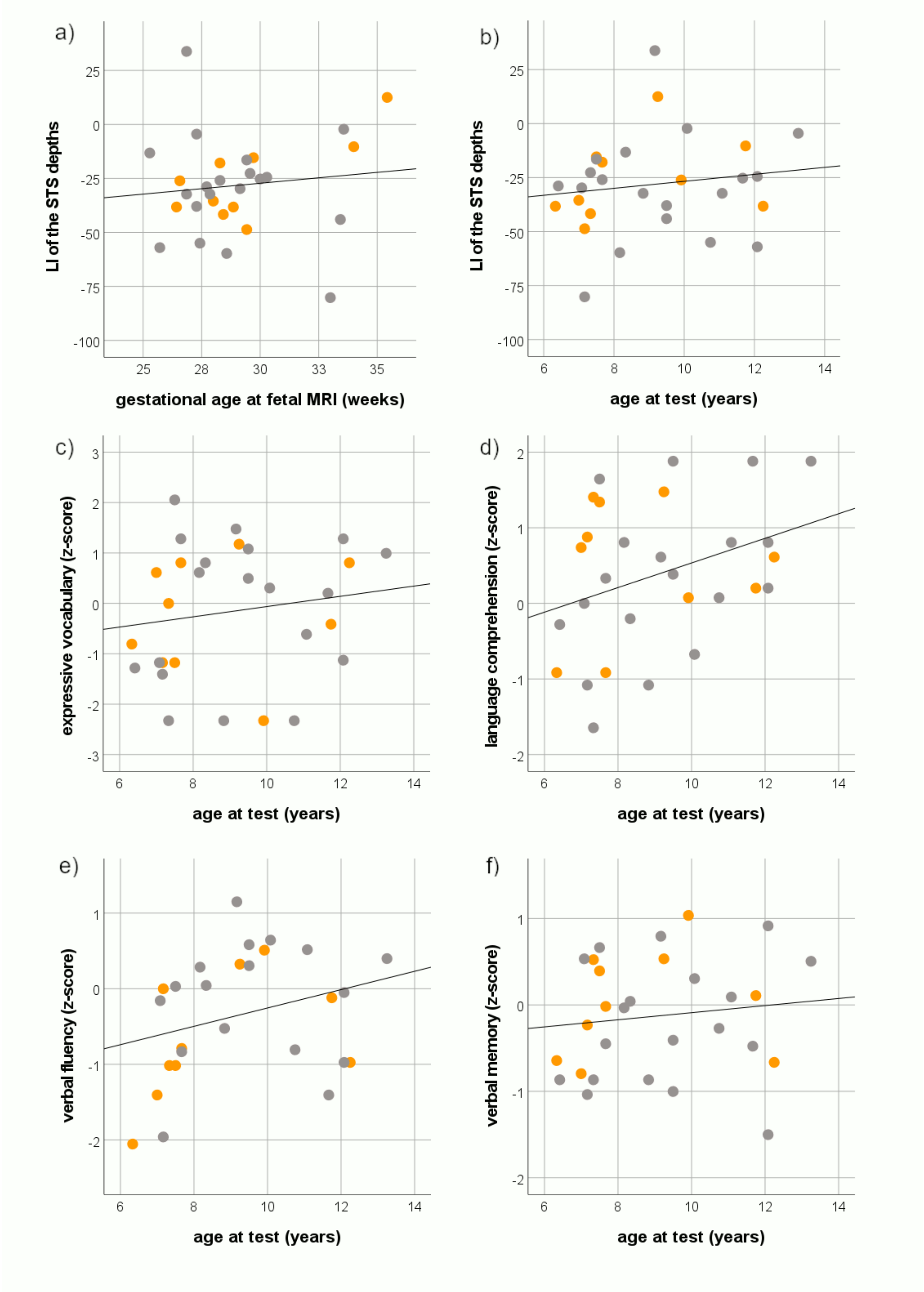

Supplementary Figure 1. Covariate relationships investigated by Pearson's correlation (n = 29). a) Correlation analysis between gestational age at fetal MRI and LI of the fetal STS depths shows no significant association between these variables (r = .12, p = .551). Thus, increasing gestational age at fetal MRI was not associated with a significant change in the asymmetry of the fetal STS depth. b) Similarily, age at test is not significantly associated with the LIs of the STS depths at fetal MRI (r = .15, p = .452). c-f) In addition, age at test is not significantly associated with expressive vocabulary, language comprehension, verbal fluency, or verbal memory (all p >. 05).

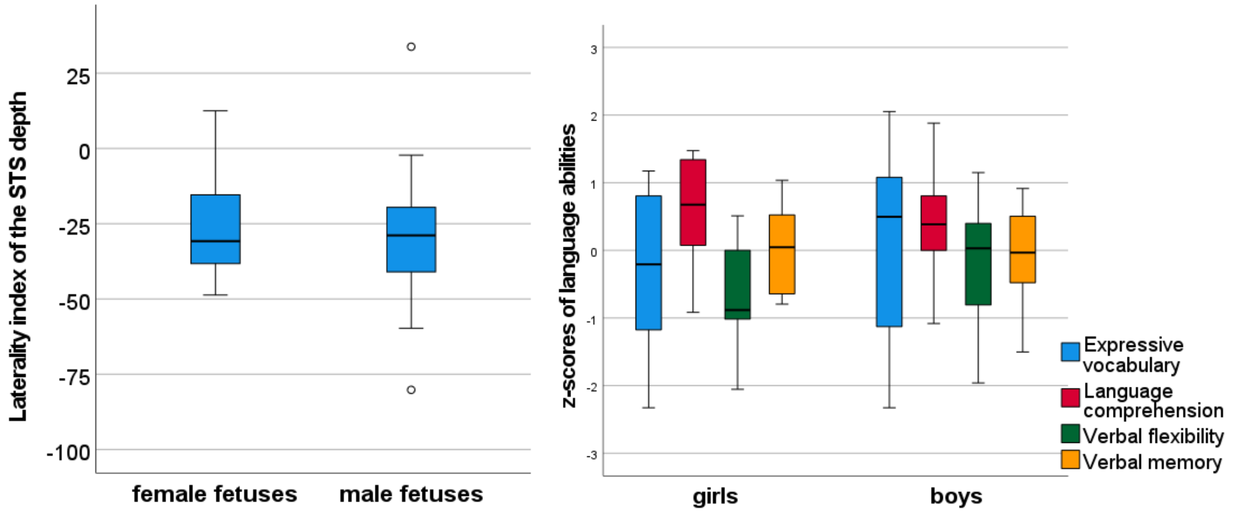

Supplementary Figure 2. Sex differences indicated by Student's t-test (n = 29). Girls and boys did not significantly differ in laterality indices of the STS depths nor in language scores (all $p > .05$), but the group of boys showed a larger data variability than the group of girls.

Additional information on the results of each variable

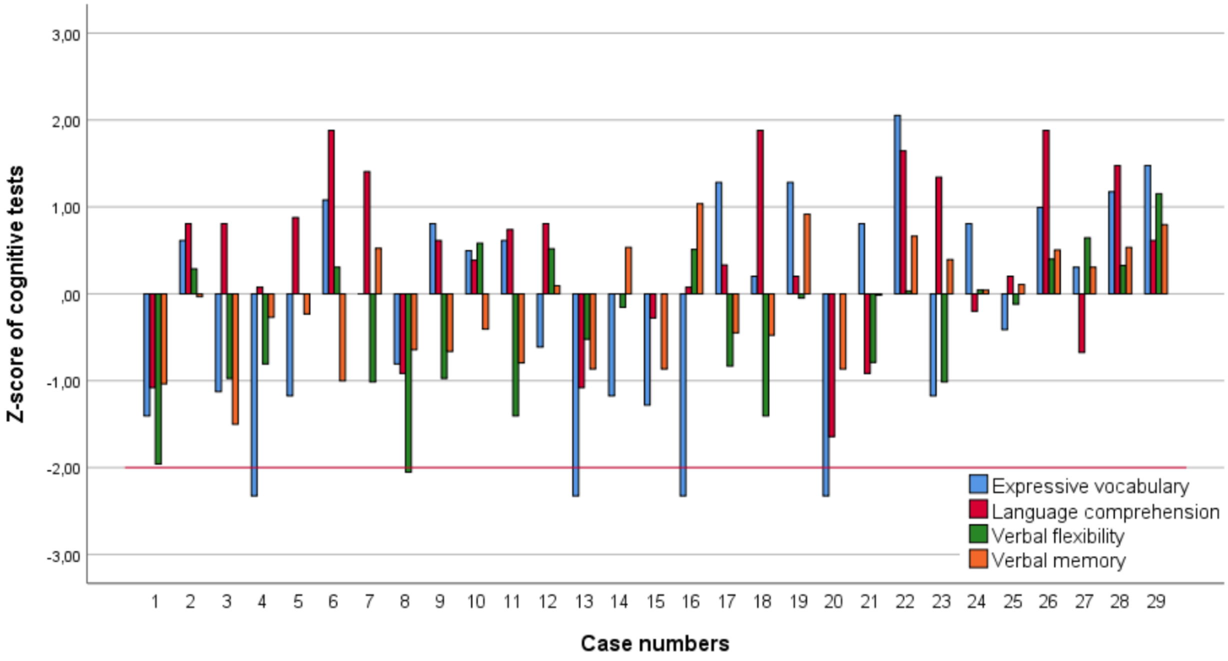

Supplementary Figure 3. Individual language profiles (n = 29). The red line indicates a z-score of -2, functions with results below this line were interpreted as reduced.

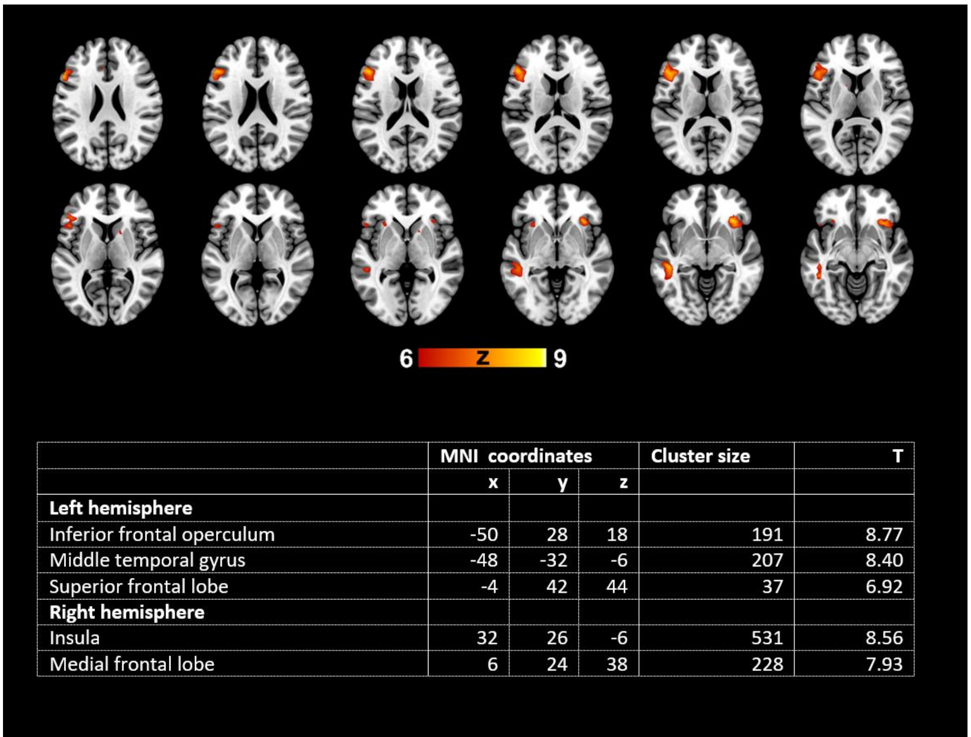

	MNI coordinates			Cluster size	T
	x	y	z		
**Left hemisphere**					
Inferior frontal operculum	-50	28	18	191	8.77
Middle temporal gyrus	-48	-32	-6	207	8.40
Superior frontal lobe	-4	42	44	37	6.92
**Right hemisphere**					
Insula	32	26	-6	531	8.56
Medial frontal lobe	6	24	38	228	7.93

Supplementary Figure 4. One-sample t-test of language activation in the whole group of participants (n = 23, $p < .05$, FWE-corrected, $k > 20$). Coordinates are given of the peak voxels in activated clusters. Group activations are depicted on a normalized T1 template, neurological convention.

Supplementary Table 3. Group analyses of language lateralization (n = 23)

	Median	25th	75th	Range
LI total	0.48	0.47	0.56	-0.59 – 0.69
LI frontal	0.45	-0.06	0.56	-0.65 – 0.75
LI temporal	0.54	-0.06	0.63	-0.68 – 0.72
LI parietal	0.27	-0.29	0.49	-0.60 – 0.65

Note: Because of non-normal distributions, LIs are presented using median, quartiles, and range

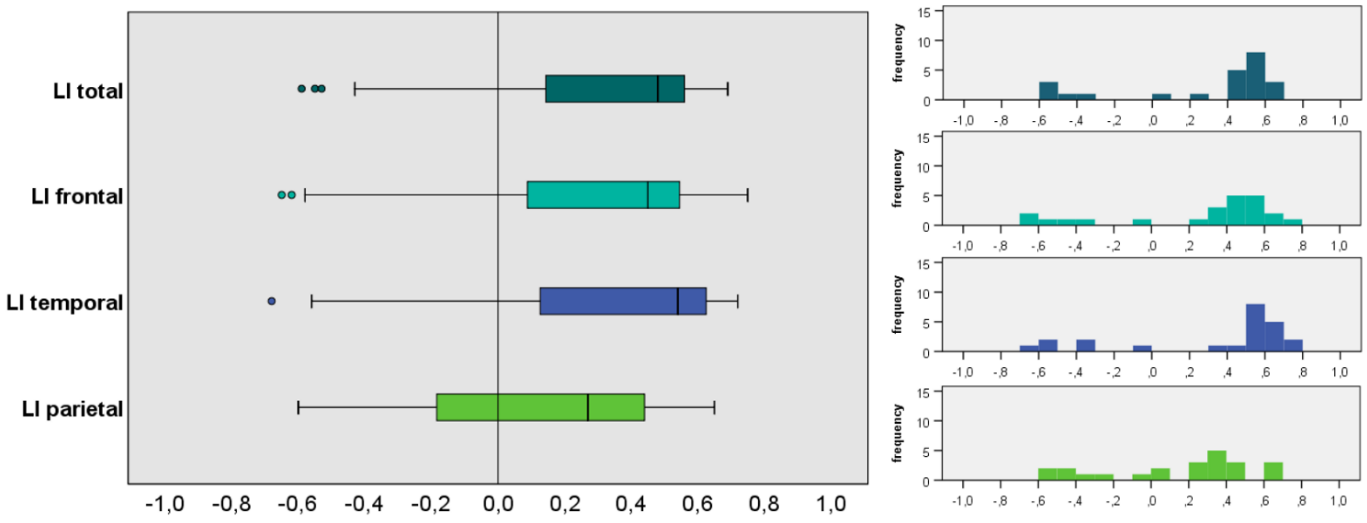

Supplementary Figure 5. Language laterality indices over the whole group of study participants (n = 23). Solid vertical lines within bars indicate medians, ° indicates asymmetric outliers.

Supplementary Table 4. Group analyses of lateralization of white matter tracts (n = 25)

	Mean (SD)	Range
**Arcuate Fasciculus**		
FA LI	1.03 (2.73)	-5.28 – 7.54
Volume LI	8.24 (35.38)	-89.32 – 78.62
Number of streams LI	15.28 (44.27)	-88.24 – 91.92
**Superior Longitudinal Fasciculus I**		
FA LI	-0.49 (2.01)	-3.88 – 5.96
Volume LI	-0.66 (20.20)	-76.83 – 36.79
Number of streams LI	5.02 (27.92)	-70.06 – 73.28
**Superior Longitudinal Fasciculus II**		
FA LI	-1.14 (2.57)	-8.47 – 2.73
Volume LI	-10.10 (26.88)	-66.53 – 34.69
Number of streams LI	-10.37 (39.31)	-89.39 – 64.29
**Superior Longitudinal Fasciculus III**		
FA LI	-1.43 (2.42)	-5.08 – 6.59
Volume LI	-11.44 (33.40)	-96.77 – 53.42
Number of streams LI	-18.31 (40.49)	-89.79 – 67.00
**Inferior longitudinal Fasciculus**		
FA LI	0.29 (2.92)	-4.85 – 6.21
Volume LI	14.82 (39.42)	-89.09 – 71.57
Number of streams LI	27.26 (42.48)	-56.68 – 87.87
**Uncinate Fasciculus**		
FA LI	0.94 (2.90)	-5.91 – 9.09
Volume LI	-3.62 (36.32)	-84.96 – 79.51
Number of streams LI	-20.59 (49.21)	-100.00 – 90.59

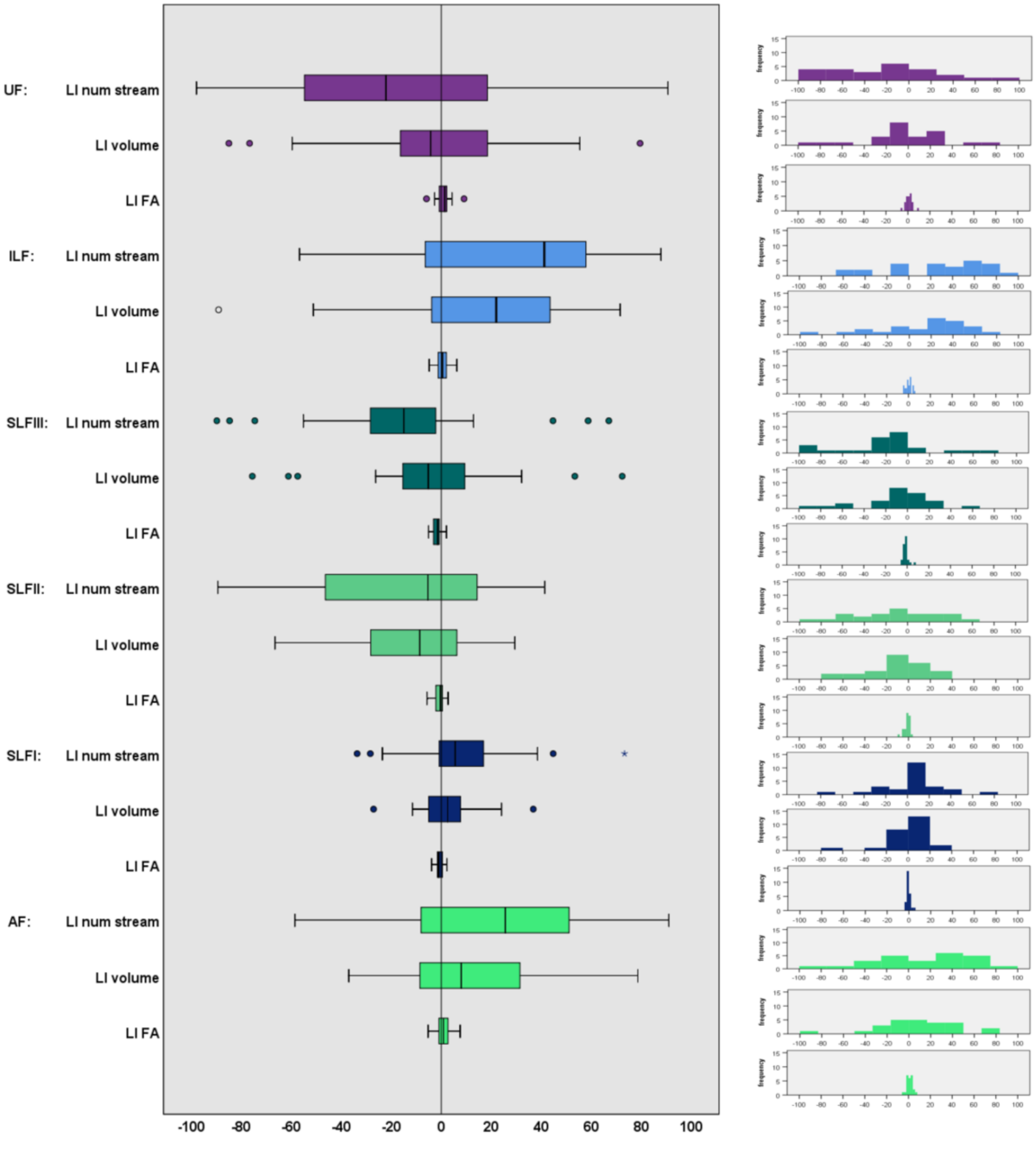

Supplementary Figure 6. Laterality indices of white matter tracts over the whole group of study participants (n = 25). UF, uncinate fasciculus; ILF, inferior longitudinal fasciculus; SLF, superior longitudinal fasciculus; AF, arcuate fasciculus; LI, laterality index; FA, fractional anisotropy. Solid vertical lines within bars indicate means, ° indicates asymmetric outliers, * indicates extreme outliers (farther than three interquartile ranges).

Additional information on the prognostic value of fetal STS depth asymmetry

Supplementary Table 5. Association between the LIs of the STS depths and language associated white matter tracts (n = 25)

	**β**	**95% CI**	**p**	**T**
Arcuate fasciculus				
Volume LI	-0.14	[-0.90, 0.49]	.538	-0.63
Left	-0.01	[-210.74, 200.03]	.957	-0.05
Right	0.17	[-116.47, 278.31]	.403	0.85
Number of streams LI	-0.17	[-1.17, 0.56]	.465	-0.74
Left	-0.02	[-48.22, 43.88]	.923	-0.10
Right	0.20	[-13.64, 37.09]	.347	0.96
Superior Longitudinal Fasciculus I				
Volume LI	-0.12	[-0.51, 0.30]	.616	-0.51
Left	0.05	[-168.44, 216.99]	.796	0.26
Right	-0.03	[-204.40, 172.80]	.863	-0.17
Number of streams LI	-0.19	[-0.77, 0.33]	.418	-0.83
Left	**0.33**	**[-78.33, -8.92]**	**.016**	**2.61**
Right	-0.29	[-74.44, 2.80]	.067	-1.93
Superior Longitudinal Fasciculus II				
Volume LI	0.24	[-0.25, 0.79]	.292	1.08
Left	0.20	[-87.65, 274.43]	.296	1.07
Right	-0.05	[-216.04, 171.05]	.811	-0.24
Number of streams LI	0.19	[-0.45, 1.08]	.400	0.86
Left	0.01	[-23.90, 24.98]	.967	0.04
Right	-0.24	[-46.93, 9.97]	.191	-1.35
Superior Longitudinal Fasciculus III				
Volume LI	0.29	[-0.26, 1.17]	.202	1.32
Left	**0.51**	**[22.46, 137.54]**	**.009***	**2.89**
Right	0.00	[-72.20, 70.75]	.983	-0.02
Number of streams LI	0.37	[--0.14, 1.39]	.103	1.70
Left	0.30	[-1.98, 11.10]	.162	1.45
Right	-0.35	[-17.66, 0.99]	.077	-1.86
Inferior Longitudinal Fasciculus				
Volume LI	-0.11	[-5.81, 11.58]	.498	0.69
Left	0.03	[-66.59, 80.67]	.844	0.20
Right	0.10	[-51.03, 84.55]	.612	0.51
Number of streams LI	-0.11	[-1.00, 0.66]	.631	-0.49
Left	-0.08	[-12.45, 6.95]	.562	-0.59
Right	0.10	[-3.98, 7.12]	.562	0.59
Uncinate Fasciculus				
Volume LI	-0.29	[-1.05, 0.18]	.157	-1.47
Left	0.11	[-89.18, 150.20]	.601	0.53
Right	0.40	[-0.49, 214.00]	.051	2.07
Number of streams LI	0.24	[-4.55, 15.69]	.190	-1.36
Left	0.10	[-31.29, 48,39]	.660	0.45
Right	0.32	[-9.71, 68.82]	.132	1.57

Note: Multiple linear regression analyses were employed, for age at test and MR device. CI, confidence intervals; LI, laterality index; Bold letters indicate $p < .05$, * indicates significance after Bonferroni correction

Additional visualizations of methods

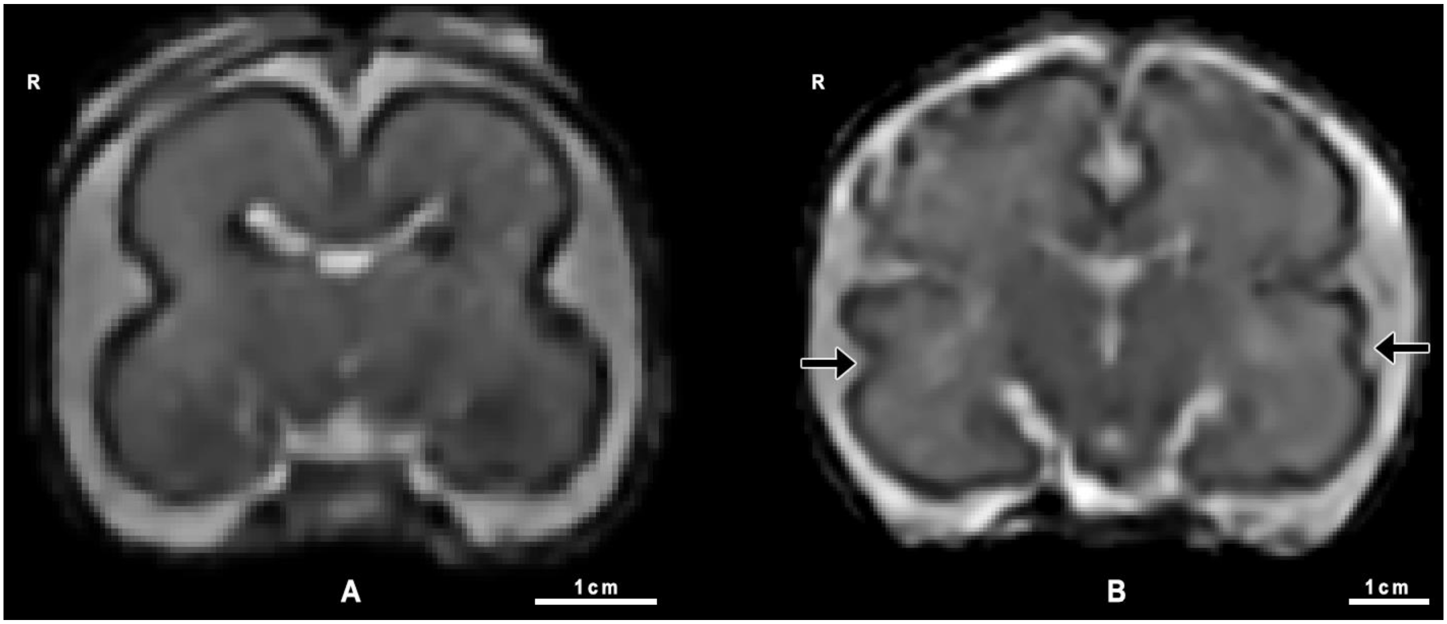

Supplementary Figure 7. Examples of superresolution-reconstructed fetal brains. A. Fetal brain at GA 21+1. STS are not developed on both sides; temporal lobes appear symmetrical. B: Typical asymmetry pattern at GA 28+3. The arrows point towards the STS, the right one is more deeply developed.

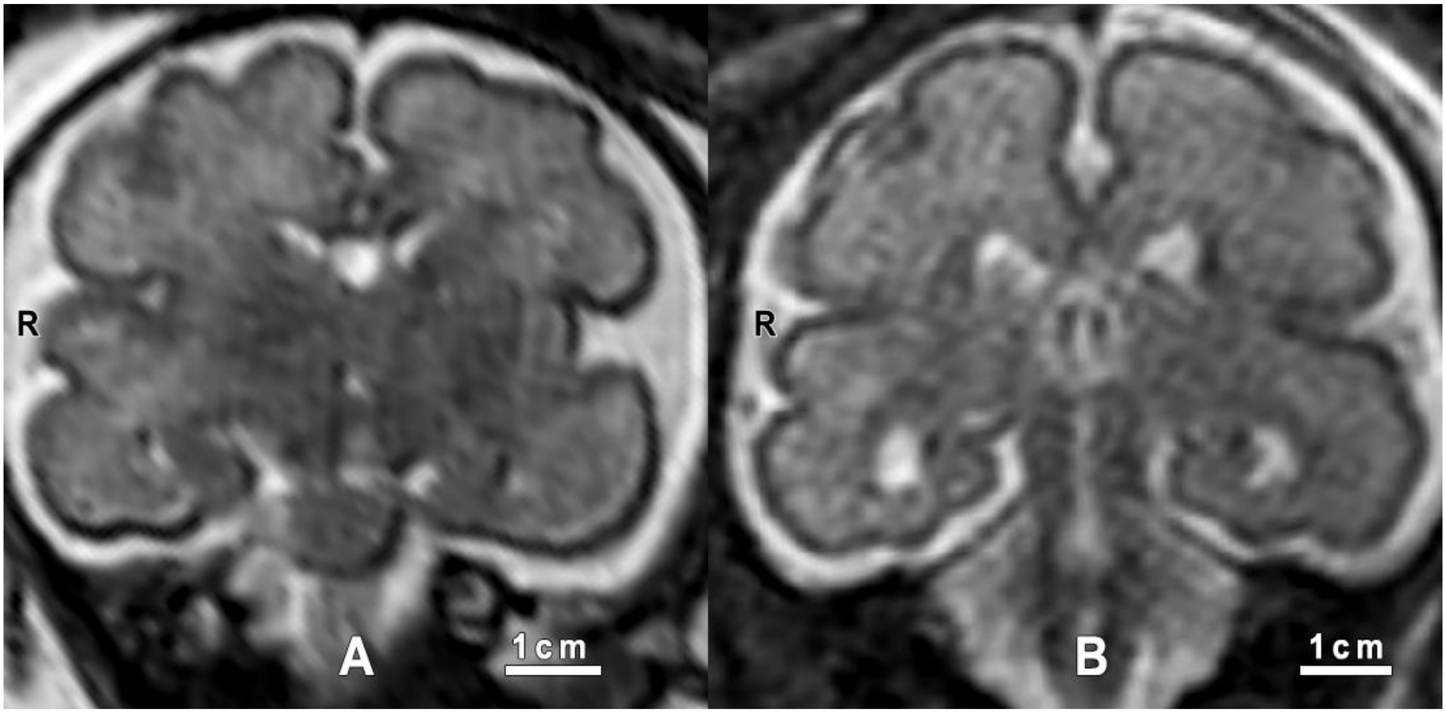

Supplementary Figure 8. Comparison of two fetuses both at GA 28+3, performed with a respective field strength of 1.5 (A) and 3 Tesla (B).

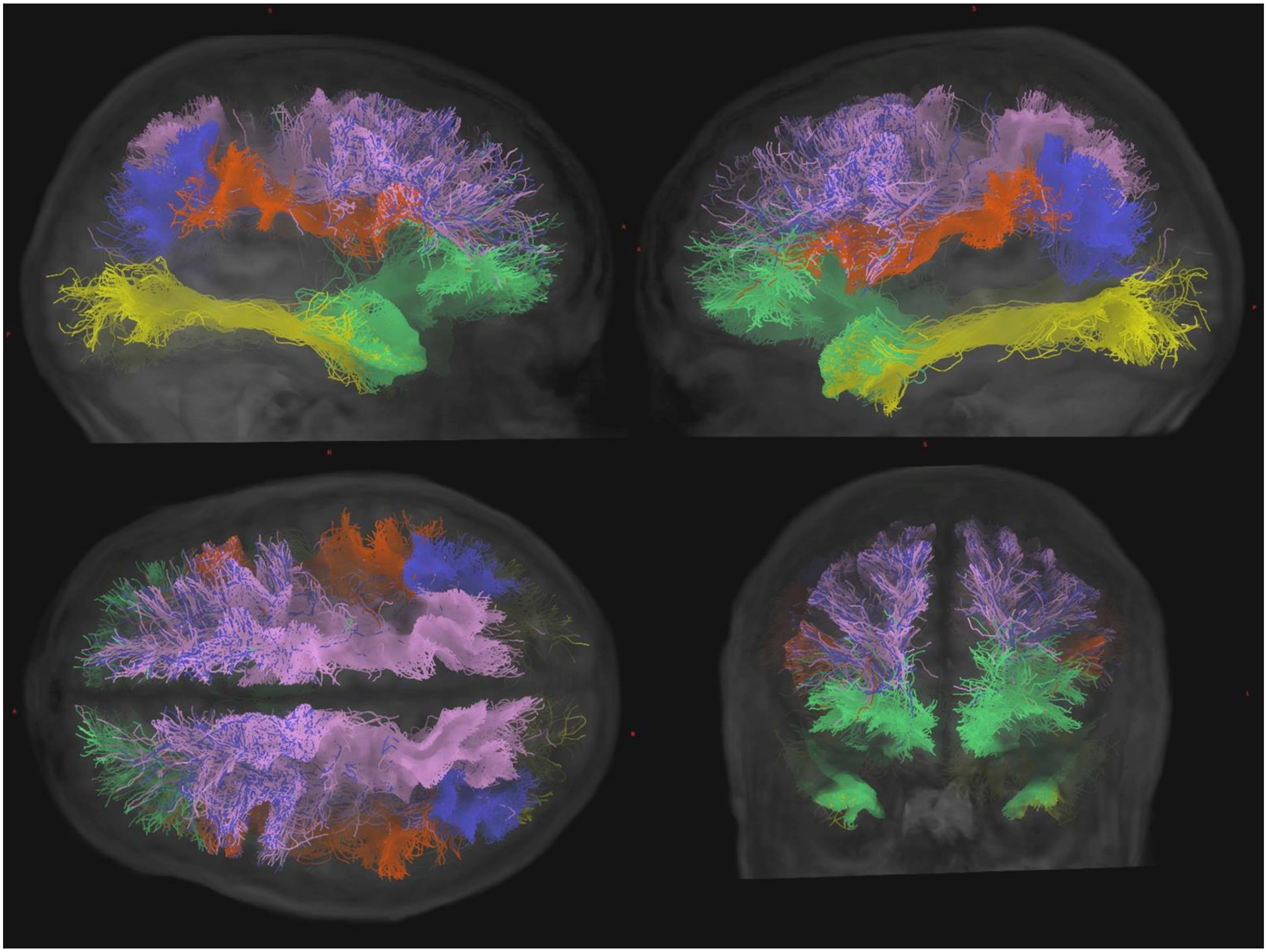

Supplementary Figure 9. Example of DTI measures in a study participant (f, 12 years old). Arcuate fasciculus, red; inferior longitudinal fasciculus, yellow; superior longitudinal fasciculus I, pink; superior longitudinal fasciculus II, purple; superior longitudinal fasciculus III, orange; uncinate fasciculus, green.

Supplementary Note 1. Alternative statistical approach to measure STS asymmetry

In addition, an alternative STS asymmetry measure was calculated accounting for possible effects of brain volume and gestational age at fetal MRI, following the approach of Bullmore et al. [1].We used multiple regression models to examine the effects of gestational age at fetal MRI and fetal brain volume on STS depths. Right and left STS depths were separately treated as dependent variables. Table 6 shows estimated regression coefficients for the explanatory terms in each model and the results of the two-tailed t-tests of the null hypothesis that the true coefficients were zero.

Gestational age at fetal MRI and brain volume at fetal MRI both had a powerful and approximately symmetrical effect on left and right STS depths. Thus, left and right STS depths were symmetrically determined by gestational age and brain volume at fetal MRI. This observation suggests that the "common denominator" assumption implicit in the LI used in the present study was justified.

Nevertheless, we followed the approach of Bullmore et al. (1995) and calculated a residual difference score (RDS = residual right STS depths – residual left STS depth). Residual right and left STS depths were generated from regression models fitted to age at fetal MRI and brain volume. Multiple linear regressions were then carried out to test if the RDS of fetal STS depth predicted language abilities 6-13 years later. Analyses were adjusted for nonverbal perceptual reasoning. A comparison of these results with the multiple linear regression results involving the LI shows that both, RDS and LI, predict language outcome to a comparable degree (Table 7).

Supplementary Table 6. Partial regression coefficients (β) for left and right STS depths on gestational age t fetal MRI and brain volume at fetal MRI

Explanatory variable	Right STS depth		Left STs depth	
	β	p	β	p
Gestational age at fetal MRI	0.645	.000	0.677	.000
Brain volume at fetal MRI	0.643	.001	0.575	.004

Supplementary Table 7. RDS and LI of STS depths predict later language abilities to a comparable degree.

	RDS			LI	
Outcome	R	p		R	p
Expressive vocabulary	**.40**	**.021***		**.43**	**.007****
Language comprehension	.15	.414		.15	.476
Verbal fluency	**.60**	**.001****		**.53**	**.002****
Verbal memory	**.54**	**.004****		**.59**	**.000****

Supplementary References

1 Bullmore, E., Brammer, M., Harvey, I. & Ron, M. Against the laterality index as a measure of cerebral asymmetry. Psychiatry Res. 61, 121-124 (1995).
