# Supplementary material for: Fetal temporal sulcus depth asymmetry has prognostic value for language development
Source: Commun Biol. 2023 Jan 27;6:109. doi: 10.1038/s42003-023-04503-z (PMC9883513; doi:10.1038/s42003-023-04503-z)
Supplement: Supplementary file 3 — Reporting Summary-New [file 42003_2023_4503_MOESM3_ESM.pdf]

nature portfolio

Corresponding author(s): Lisa Bartha-Doering
Last updated by author(s): Jan 10, 2023

Reporting Summary

Nature Portfolio wishes to improve the reproducibility of the work that we publish. This form provides structure for consistency and transparency in reporting. For further information on Nature Portfolio policies, see our Editorial Policies and the Editorial Policy Checklist.

Statistics

For all statistical analyses, confirm that the following items are present in the figure legend, table legend, main text, or Methods section.

n/a	Confirmed	
☐	☒	The exact sample size (n) for each experimental group/condition, given as a discrete number and unit of measurement
☐	☒	A statement on whether measurements were taken from distinct samples or whether the same sample was measured repeatedly
☐	☒	The statistical test(s) used AND whether they are one- or two-sided<br>*Only common tests should be described solely by name; describe more complex techniques in the Methods section.*
☐	☒	A description of all covariates tested
☐	☒	A description of any assumptions or corrections, such as tests of normality and adjustment for multiple comparisons
☐	☒	A full description of the statistical parameters including central tendency (e.g. means) or other basic estimates (e.g. regression coefficient) AND variation (e.g. standard deviation) or associated estimates of uncertainty (e.g. confidence intervals)
☐	☒	For null hypothesis testing, the test statistic (e.g. F, t, r) with confidence intervals, effect sizes, degrees of freedom and P value noted<br>*Give P values as exact values whenever suitable.*
☒	☐	For Bayesian analysis, information on the choice of priors and Markov chain Monte Carlo settings
☒	☐	For hierarchical and complex designs, identification of the appropriate level for tests and full reporting of outcomes
☐	☒	Estimates of effect sizes (e.g. Cohen's d, Pearson's r), indicating how they were calculated

Our web collection on statistics for biologists contains articles on many of the points above.

Software and code

Policy information about availability of computer code

Data collection	MRI was performed using Ingenia, Philips Medical Systems, Siemens Medical Solutions, Erlangen Germany, and Philips Elition 3 Tesla. FMRI stimuli were presented with Nordic NeuroLab (https://www.nordicneurolab.com/product/fmri-acquisition)
Data analysis	Analyses were conducted using software and toolboxes as described in “Methods” (SPM 12: https://www.fil.ion.ucl.ac.uk/spm/software/spm12/; Freesurver: https://surfer.nmr.mgh.harvard.edu/; SPSS Statistics 28: https://www.ibm.com/de-de/products/spss-statistics)

For manuscripts utilizing custom algorithms or software that are central to the research but not yet described in published literature, software must be made available to editors and reviewers. We strongly encourage code deposition in a community repository (e.g. GitHub). See the Nature Portfolio guidelines for submitting code & software for further information.

Data

Policy information about availability of data

All manuscripts must include a data availability statement. This statement should provide the following information, where applicable:

- Accession codes, unique identifiers, or web links for publicly available datasets
- A description of any restrictions on data availability
- For clinical datasets or third party data, please ensure that the statement adheres to our policy

Data of the study are available at https://osf.io/surv5/?view_only=7510f6f2114844b0b6cda19eb9f441a0

Human research participants

Policy information about studies involving human research participants and Sex and Gender in Research.

Reporting on sex and gender	We included 14 girls and 24 boys in this study. Sex was reported and included in all analyses.
Population characteristics	The sample consisted of 38 children whose mothers were transferred to fetal MRI diagnostics due to clinical reasons 6-13 years ago and whose fetal MRIs were subsequently diagnosed as normal. Mean age of the children at fetal MRI was 27.88 weeks (SD 3.57), at test mean age was 8.85 years (SD 1.98).
Recruitment	We contacted the women who have had fetal MRI with normal findings 6-13 years ago per mail and phone and invite them and their child to participate in our study.
Ethics oversight	Ethics Committee of the Medical University of Vienna (Nr. 1083/2015)

Note that full information on the approval of the study protocol must also be provided in the manuscript.

Field-specific reporting

Please select the one below that is the best fit for your research. If you are not sure, read the appropriate sections before making your selection.

☒ Life sciences ☐ Behavioural & social sciences ☐ Ecological, evolutionary & environmental sciences

For a reference copy of the document with all sections, see nature.com/documents/nr-reporting-summary-flat.pdf

Life sciences study design

All studies must disclose on these points even when the disclosure is negative.

Sample size	The present project was the first to study the possible association of prenatal STS asymmetry, later language localization, and later language abilities. However, there were group studies on the association of language lateralization and cortical asymmetry. Greve et al. (2013) investigated 55 healthy adults with fMRI for language lateralization and MRI for gray matter volume estimation and found a correlation between a language fMRI paradigm and asymmetry of the insula of rho .38. Thus, to find a correlation between the language lateralization index and the index of structural asymmetry with a probability of Type I Error (α = 0.05) and the power ($1 - \beta$) of 0.8, 40 subjects would be needed. Due to this sample size calculation, our goal was to include 40 children in our study.
Data exclusions	No data was excluded from the analysis
Replication	Not applicable
Randomization	Not applicable
Blinding	Not applicable

Reporting for specific materials, systems and methods

We require information from authors about some types of materials, experimental systems and methods used in many studies. Here, indicate whether each material, system or method listed is relevant to your study. If you are not sure if a list item applies to your research, read the appropriate section before selecting a response.

Materials & experimental systems

n/a	Involved in the study	
☒	☐	Antibodies
☒	☐	Eukaryotic cell lines
☒	☐	Palaeontology and archaeology
☒	☐	Animals and other organisms
☐	☒	Clinical data
☐	☐	Dual use research of concern

Methods

n/a	Involved in the study	
☒	☐	ChIP-seq
☒	☐	Flow cytometry
☐	☒	MRI-based neuroimaging

Clinical data

Policy information about clinical studies

All manuscripts should comply with the ICMJE guidelines for publication of clinical research and a completed CONSORT checklist must be included with all submissions.

Clinical trial registration	This study was preregistered in the in the German Clinical Trials Register (DRKS00010582)
Study protocol	The study protocol can be found in the in the German Clinical Trials Register (DRKS00010582)
Data collection	Mothers were recruited by mail and phone. MR imaging and cognitive testing were performed within one or two weeks from each other.Time between recruitment and test was about 1 to 3 months.
Outcomes	Predefiniton of outcomes in DRKS00010582:<br>1) a positive correlation between prenatal structural lateralization in the planum temporale and child language localization.<br>2) a positive correlation between prenatal structural asymmetry in the planum temporale and language-related fiber connections in the infant brain.<br>3) a correlation between prenatal anatomical asymmetry in the temporal planum and later language skills, in which prenatal left-sided temporal planum asymmetry is significantly associated with better language skills.<br>4) specific markers in fetal MRI for later language skills.

Dual use research of concern

Policy information about dual use research of concern

Hazards

Could the accidental, deliberate or reckless misuse of agents or technologies generated in the work, or the application of information presented in the manuscript, pose a threat to:

No	Yes	
☒	☐	Public health
☒	☐	National security
☒	☐	Crops and/or livestock
☒	☐	Ecosystems
☒	☐	Any other significant area

Experiments of concern

Does the work involve any of these experiments of concern:

No	Yes	
☒	☐	Demonstrate how to render a vaccine ineffective
☒	☐	Confer resistance to therapeutically useful antibiotics or antiviral agents
☒	☐	Enhance the virulence of a pathogen or render a nonpathogen virulent
☒	☐	Increase transmissibility of a pathogen
☒	☐	Alter the host range of a pathogen
☒	☐	Enable evasion of diagnostic/detection modalities
☒	☐	Enable the weaponization of a biological agent or toxin
☒	☐	Any other potentially harmful combination of experiments and agents

Magnetic resonance imaging

Experimental design

Design type	Standard structural neuroimaging (T1, T2), task-based fMRI (EPI) Fetal MRI: T2-weighted turbo spin-echo sequences<br>FMRI: MPRAGE, T1-weighted, EPI (task-based)<br>DTI: T1-weighted
Design specifications	FMRI: In the auditory description definition condition, the participants heard the definition of an object followed by a noun and were instructed to press a button each time the definition truly described the noun. The control condition consisted of reverse speech, with some items additionally containing a pure tone at the end. The participants were instructed to press the button each time he/she heard the tone. Seventy percent of items were correct targets in both conditions. True and false descriptions were pseudorandomly distributed.<br>Three different, age-adjusted versions of the fMRI paradigm were available (7–9 years old, 10–12 years old, 13–16 years old). The difficulty levels were achieved by manipulating linguistic criteria, including word frequency, word length, and

word complexity according to normative word data. We used a block design composed of five language task blocks alternating with five control task blocks. Each block lasted for 40 s and consisted of 10 sentences presented every 4 s. Total fMRI scan time was 6 min 40.

Behavioral performance measures: Performance in the scanner was monitored by button-press. Task accuracy was evaluated by the overall accuracy in the language task and the control task separately.

Acquisition

Imaging type(s): structural, functional, diffusion

Field strength: T1, T2, EPI

Sequence & imaging parameters:

Fetal MRI: T2-weighted turbo spin-echo sequences: in-plane resolution, 0.62/0.62–1.17/1.17mm; slice thickness, 2.0–4.5 mm; matrix size, 256 x 256; field of view, 200–230 mm; relaxation time, ≤20000 ms; echo time, 100–140 ms

3D structural MRI: MPRAGE, T1-weighted, TE/TR _ 4.21/2300ms, inversion time 900ms, with a matrix size of 240 x 256 x 160, voxel size 1 x 1 x 1.10mm, flip angle 9°

fMRI: EPI: FOV of 210 mm, voxel size 2.1 x 2.1 x 4 mm, 20 slices with a gap of 25 percent were aligned parallel to the AC-PC plane; repetition time (TR) was 2000 msec, echo time (TE) 42 msec, flip angle 90 degrees.

DTI: T1: TE/TR _ 4.21/2300ms, inversion time 900ms, with a matrix size of 240 x 256 x 160, voxel size 1 x 1 x 1.10mm, flip angle 9°, b-values of 0 and 1000s/mm²; 30 gradient encoding directions; acquired voxel size 2 x 2 x 2 mm; TR = 8000ms; TE = 83ms

Area of acquisition: whole brain

Diffusion MRI: ☒ Used ☐ Not used

Parameters: T1: TE/TR _ 4.21/2300ms, inversion time 900ms, with a matrix size of 240 x 256 x 160, voxel size 1 x 1 x 1.10mm, flip angle 9°, b-values of 0 and 1000s/mm²; 30 gradient encoding directions; acquired voxel size 2 x 2 x 2 mm; TR = 8000ms; TE = 83ms

Preprocessing

Preprocessing software: SPM 12 (Wellcome Department of Cognitive Neurology, London, UK)

Normalization: Each subject's anatomical image was segmented with the customized priors and the customized T1 template. After coregistration, the derived spatial normalization parameters were used to normalize the functional volumes.

Normalization template: Customized prior probability maps and a customized T1 template, matched to age and gender composition of the study group, were created by employing the Template-O-Matic (TOM) toolbox (Wilke, Holland, Altaye, & Gaser, 2008). This approach employs the general linear model and is based on pediatric imaging data from the NIH study on healthy brain development (Evans & Brain Development Cooperative, 2006). It statistically isolates the influence of age or gender on brain structure and thus produces high-quality matched templates for our pediatric study population.

Noise and artifact removal: Frame-to-frame displacement between successive volumes was estimated by calculating the Euclidian distance from the translational parameters obtained from the realignment. A regressor modeling residual movement-related variance (translational and rotational movement) was included in the model as a covariate of no interest.

Volume censoring: N/A

Statistical modeling & inference

Model type and settings: group map of language activations: second level one-sample t-test, FWE-corrected
association of language activations with LI of STS: second level multiple regression, adjusted for age and MR device, FWE-corrected

Effect(s) tested: see above

Specify type of analysis: ☒ Whole brain ☐ ROI-based ☐ Both

Statistic type for inference (See Eklund et al. 2016): voxel-wise

Correction: FWE-corrected

Models & analysis

n/a	Involved in the study
☒	☐ Functional and/or effective connectivity
☒	☐ Graph analysis
☒	☐ Multivariate modeling or predictive analysis
